# Supplementary material for: Roles of Species-Specific Legumains in Pathogenicity of the Pinewood Nematode Bursaphelenchus xylophilus
Source: Int J Mol Sci. 2022 Sep 9;23(18):10437. doi: 10.3390/ijms231810437 (PMC9499627; doi:10.3390/ijms231810437)
Supplement: Supplementary file 1 [file ijms-23-10437-s001.zip › ijms-1817838-supplementary.pdf]

Figure S1: Relationship of predicted gene number to genome size (a) and to assembled scaffold N50 (b) in PPN genomes.
Figure S2: Phylogeny and structures of nematode legumains and plants VPEs.
Figure S3: Global alignment of legumain sequences by MAFFT online server.
Figure S4: Structures and comparisons of legumains.
Figure S5: Necrotic lesions on different types of *Arabidopsis* plants after infiltration of a 10-µl of FB1 into each leaf for five days.
Figure S6: The heatmap of DEGs in leaves of different types of *Arabidopsis* plants and RT-qPCR verification.
Figure S7: Comparison of BxCN10334 and BxCA10290.
Table S1: General features of *Bursaphelenchus xylophilus* and *B. mucronatus* genomes.
Table S2: List of nematode genomes used in this study.
Table S3: Summary of predicted genes encoding peptidases in 26 plant-parasitic nematode genomes;
Table S4: Summary of predicted genes encoding secretory peptidases and inhibitors in 26 plant-parasitic nematode genomes.
Table S5: Information of legumain-coding genes in *Bursaphelenchus* genomes.
Table S6: Results of statistical analysis of lesion sizes of different *Arabidopsis* plants.
Table S7: List of 59 DEGs with identical expression patterns in *Arabidopsis* transcriptomes of the wild-type and the transgenic lines with two *B. xylophilus*-specific legumains, compared with *Δγvpe* mutant.
Table S8: Enriched GO terms of the 59 DEGs shared in *Arabidopsis* transcriptomes of the wild-type and the transgenic lines with two *B. xylophilus*-specific legumains.
Table S9: List of additional 1333 DEGs with identical expression patterns in *Arabidopsis* transcriptomes of the transgenic lines with two *B. xylophilus*-specific legumains, compared with *Δγvpe* mutant.
Table S10: Enriched GO terms of the additional 1333 DEGs shared in *Arabidopsis* transcriptomes of the transgenic lines with two *B. xylophilus*-specific legumains.
Table S11: Details of DNA library construction and sequence data output.
Table S12: Sequences of primer-pairs used in this study.
