# Supplementary figures and images for: Roles of Species-Specific Legumains in Pathogenicity of the Pinewood Nematode Bursaphelenchus xylophilus
Source: Int J Mol Sci. 2022 Sep 9;23(18):10437. doi: 10.3390/ijms231810437 (PMC9499627; doi:10.3390/ijms231810437)

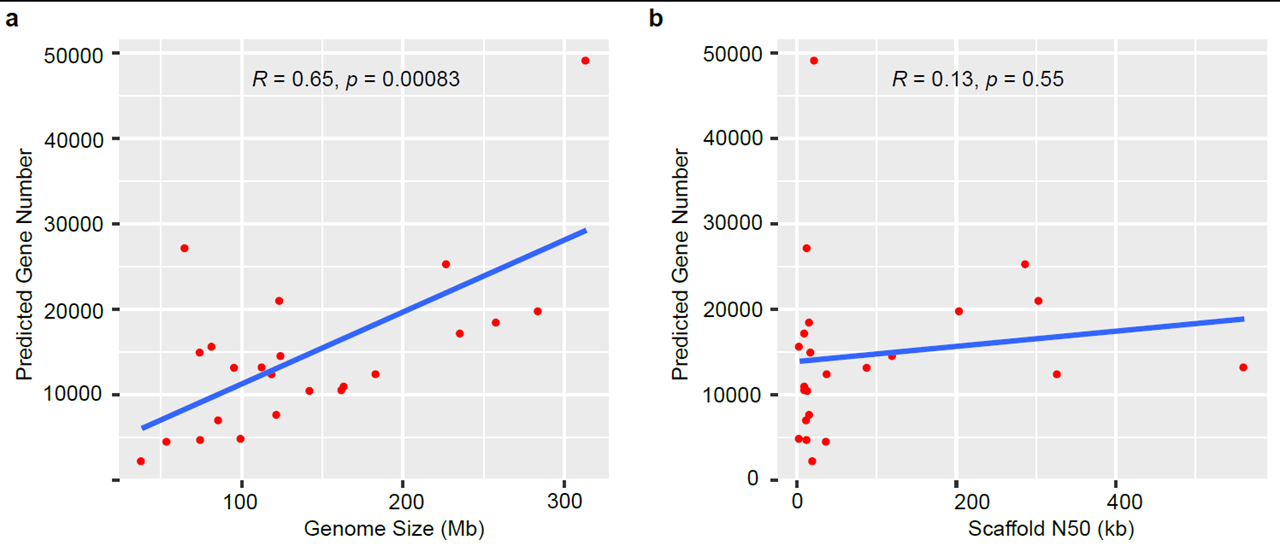

Supplement: Supplementary file 1 [file ijms-23-10437-s001.zip › supplementary Figure S1.tif]

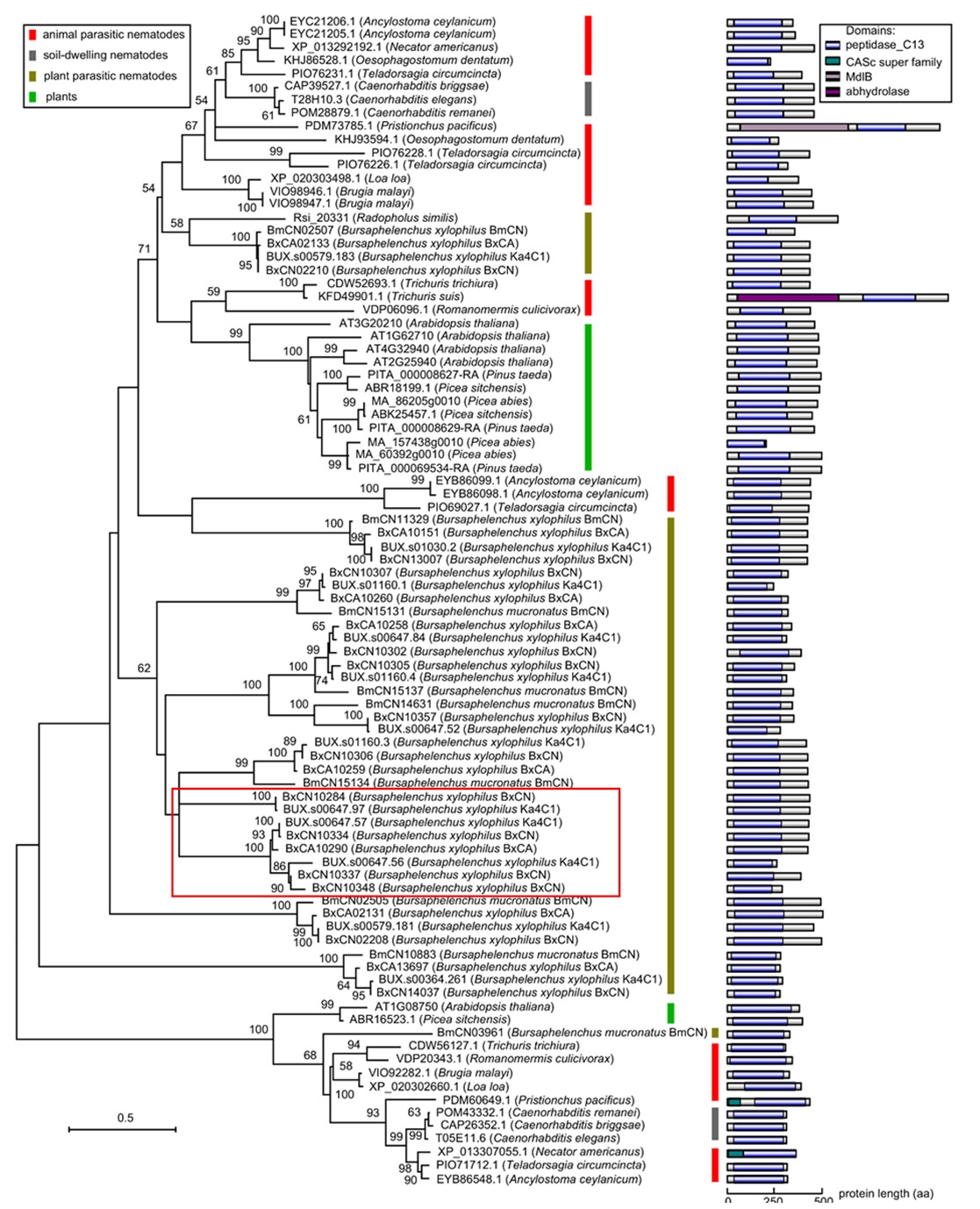

Supplement: Supplementary file 1 [file ijms-23-10437-s001.zip › supplementary Figure S2.tif]

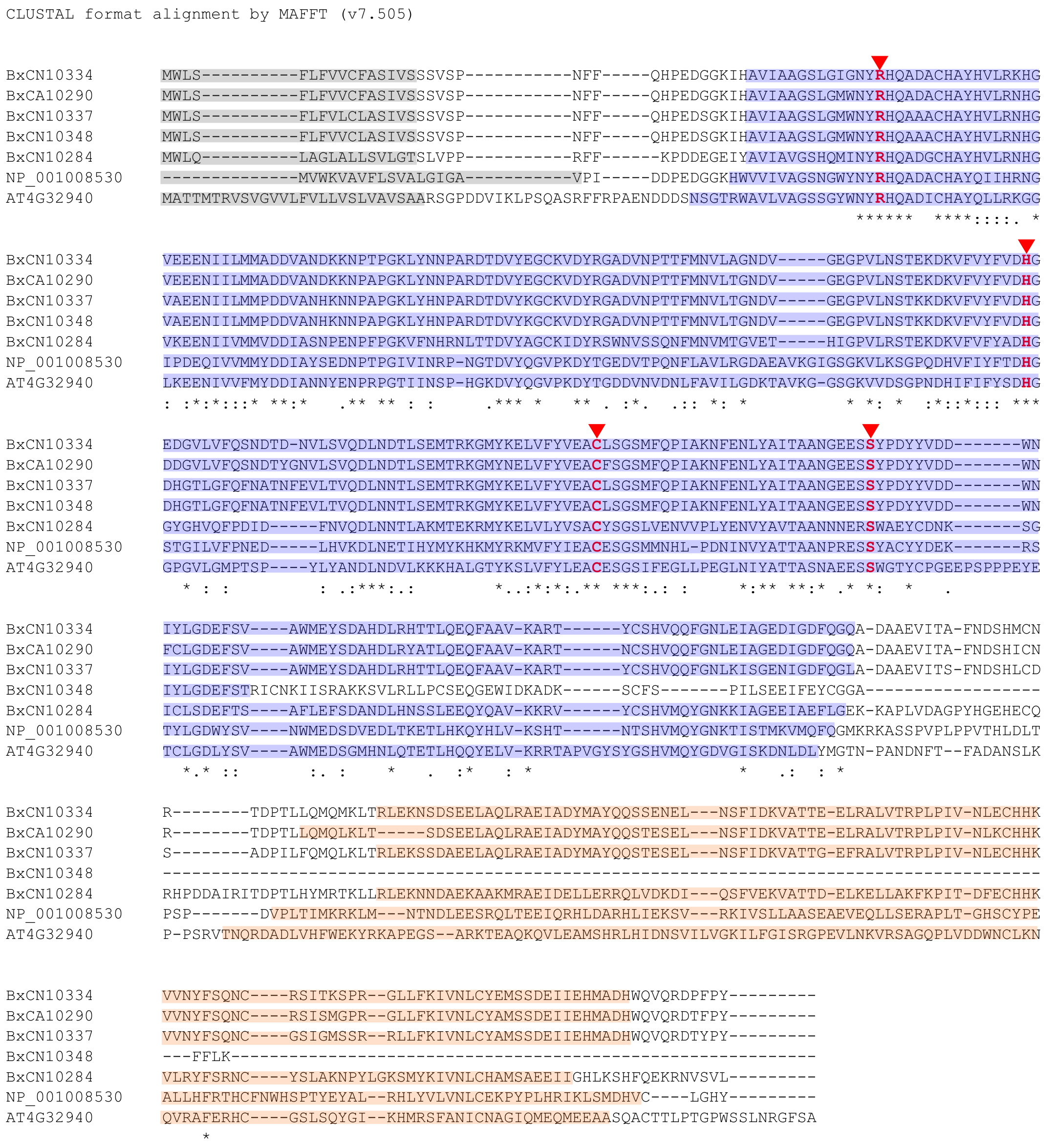

Supplement: Supplementary file 1 [file ijms-23-10437-s001.zip › supplementary Figure S3.tiff]

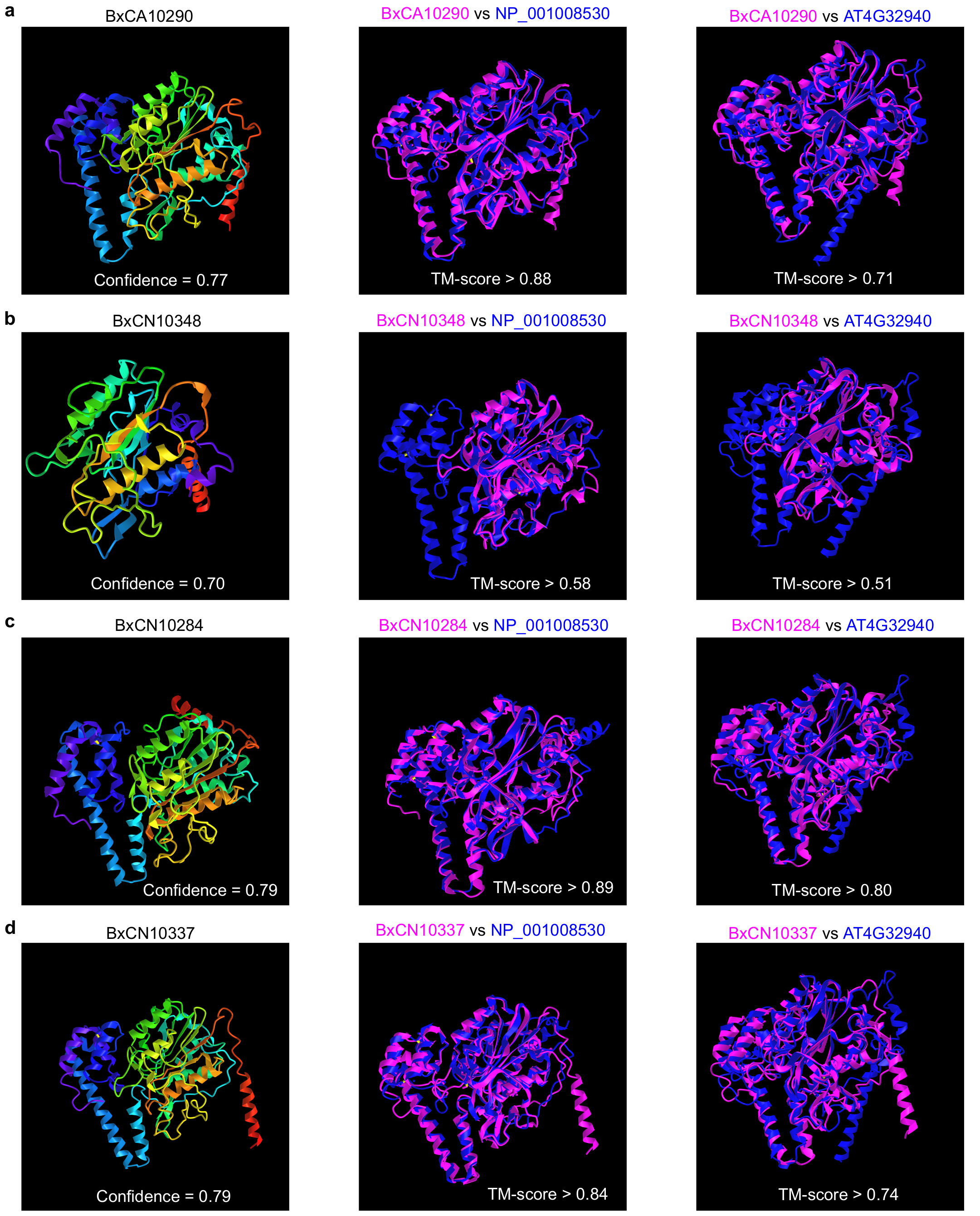

Supplement: Supplementary file 1 [file ijms-23-10437-s001.zip › supplementary Figure S4.tiff]

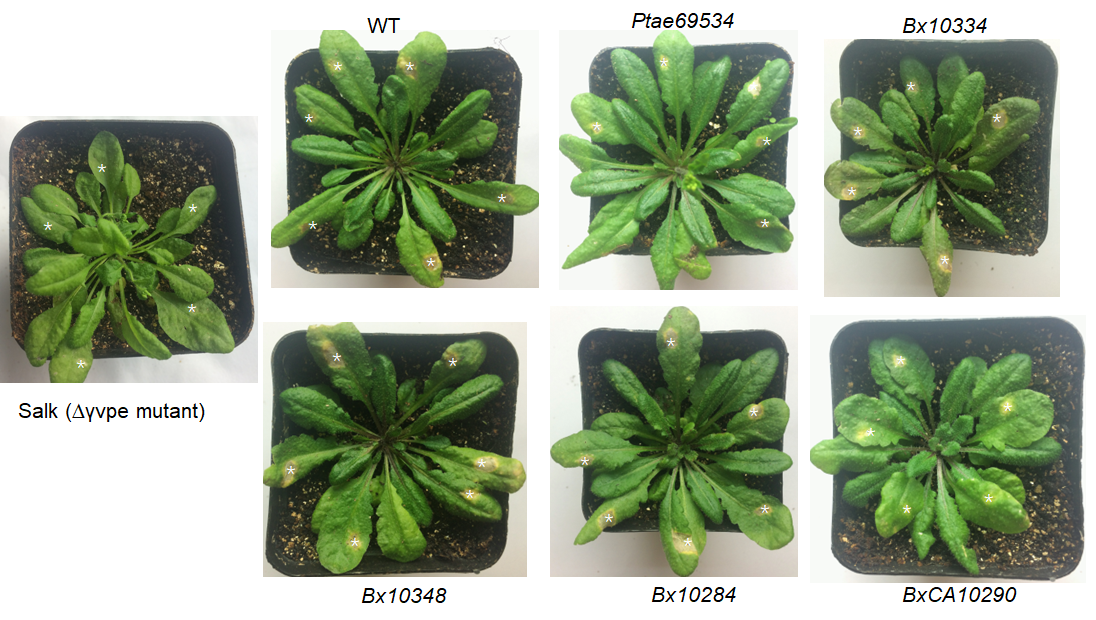

Supplement: Supplementary file 1 [file ijms-23-10437-s001.zip › supplementary Figure S5.tif]

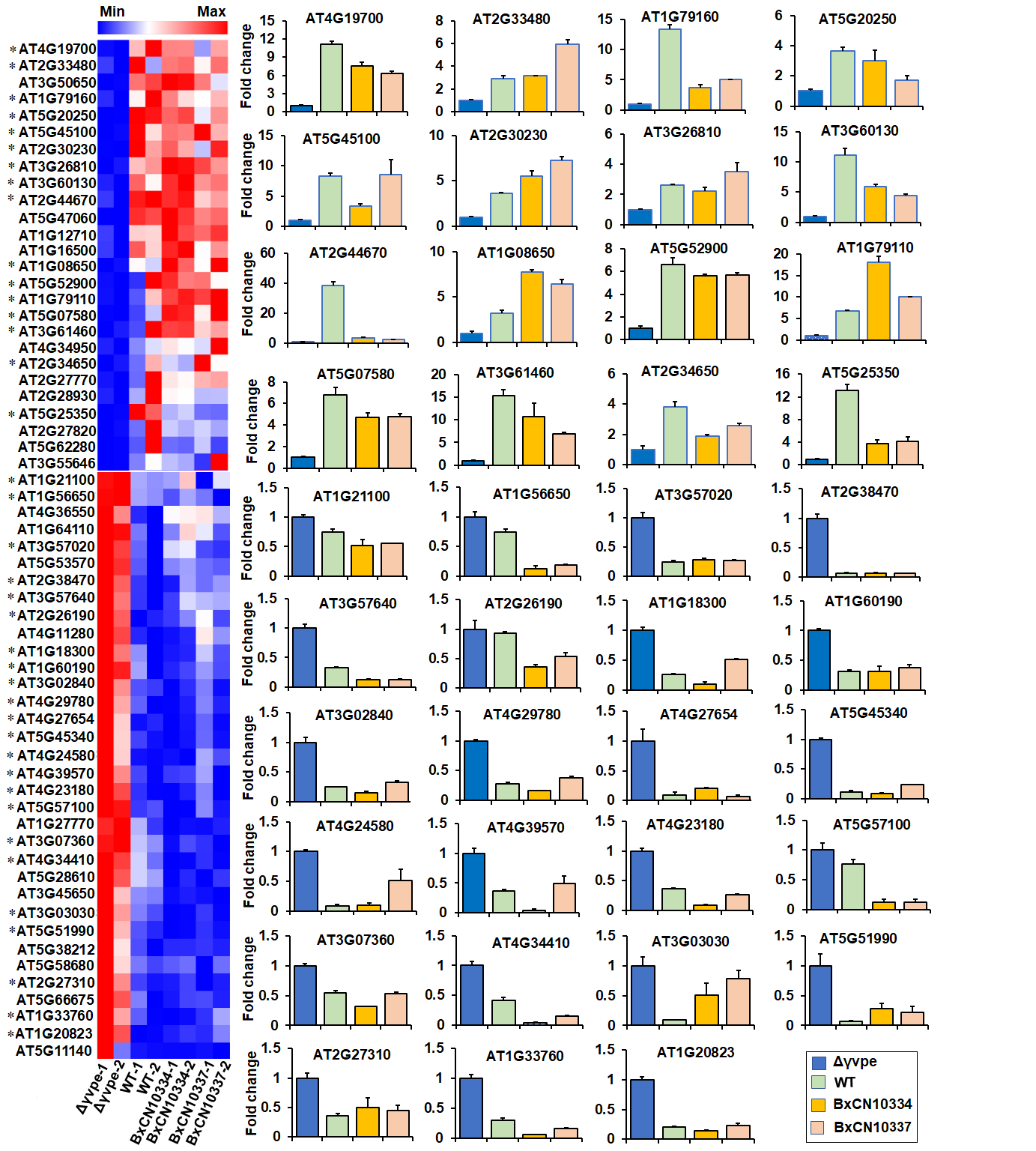

Supplement: Supplementary file 1 [file ijms-23-10437-s001.zip › supplementary Figure S6.tif]

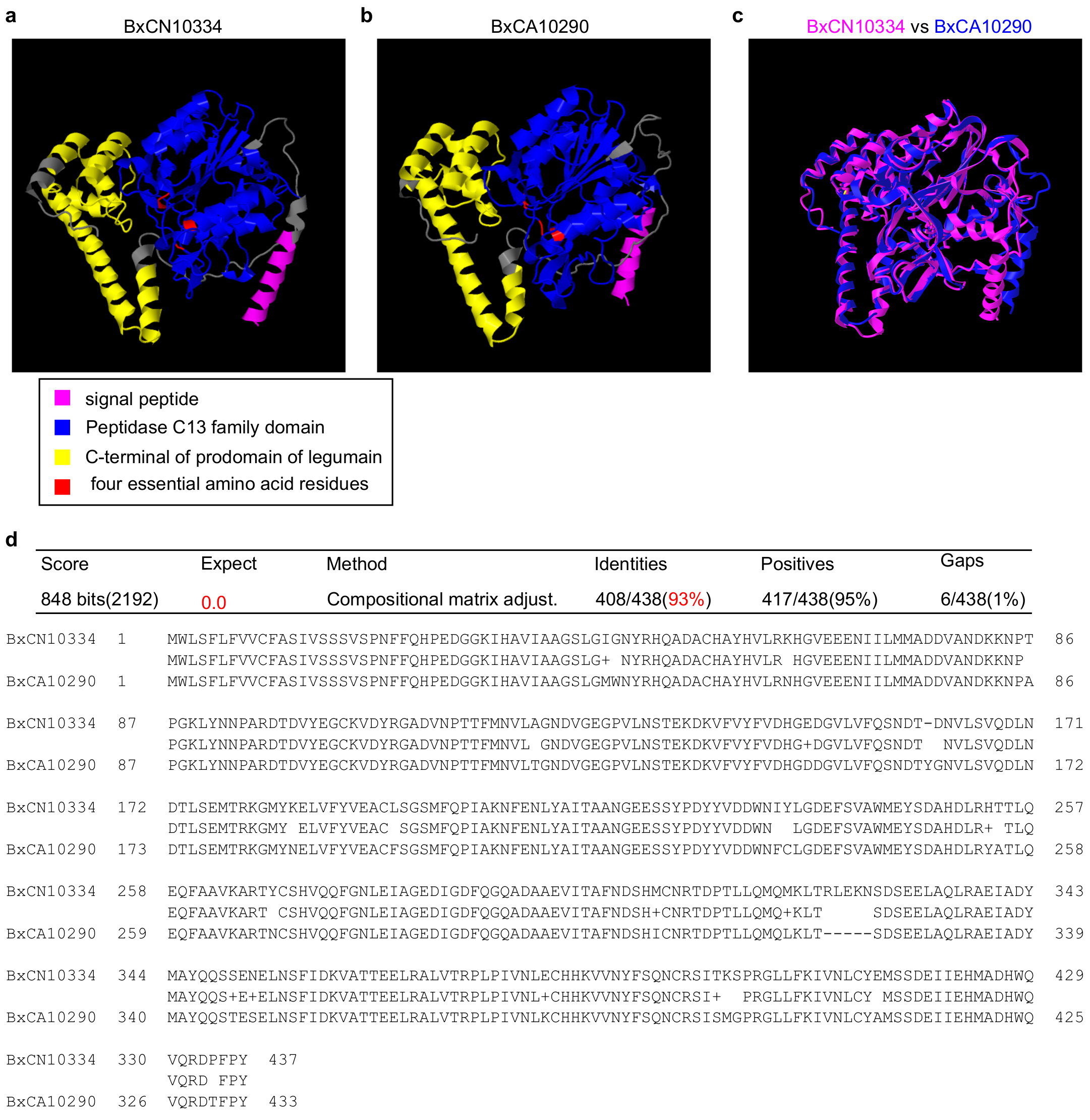

Supplement: Supplementary file 1 [file ijms-23-10437-s001.zip › supplementary Figure S7.tiff]
